# Development and Applications of a High Throughput Genotyping Tool for Polyploid Crops: Single Nucleotide Polymorphism (SNP) Array

**DOI:** 10.3389/fpls.2018.00104

**Published:** 2018-02-06

**Authors:** Qian You, Xiping Yang, Ze Peng, Liping Xu, Jianping Wang

**Affiliations:** ^1^Key Laboratory of Sugarcane Biology and Genetic Breeding Ministry of Agriculture, Fujian Agriculture and Forestry University, Fuzhou, China; ^2^Agronomy Department, University of Florida, Gainesville, FL, United States; ^3^Plant Molecular and Cellular Biology Program, Genetics Institute, University of Florida, Gainesville, FL, United States; ^4^Key Laboratory of Genetics, Breeding and Multiple Utilization of Crops, Ministry of Education, Fujian Provincial Key Laboratory of Haixia Applied Plant Systems Biology, Center for Genomics and Biotechnology, Fujian Agriculture and Forestry University, Fuzhou, China

**Keywords:** polyploidy, SNP, SNP array, genotype calling, high throughput genotyping

## Abstract

Polypoid species play significant roles in agriculture and food production. Many crop species are polyploid, such as potato, wheat, strawberry, and sugarcane. Genotyping has been a daunting task for genetic studies of polyploid crops, which lags far behind the diploid crop species. Single nucleotide polymorphism (SNP) array is considered to be one of, high-throughput, relatively cost-efficient and automated genotyping approaches. However, there are significant challenges for SNP identification in complex, polyploid genomes, which has seriously slowed SNP discovery and array development in polyploid species. Ploidy is a significant factor impacting SNP qualities and validation rates of SNP markers in SNP arrays, which has been proven to be a very important tool for genetic studies and molecular breeding. In this review, we (1) discussed the pros and cons of SNP array in general for high throughput genotyping, (2) presented the challenges of and solutions to SNP calling in polyploid species, (3) summarized the SNP selection criteria and considerations of SNP array design for polyploid species, (4) illustrated SNP array applications in several different polyploid crop species, then (5) discussed challenges, available software, and their accuracy comparisons for genotype calling based on SNP array data in polyploids, and finally (6) provided a series of SNP array design and genotype calling recommendations. This review presents a complete overview of SNP array development and applications in polypoid crops, which will benefit the research in molecular breeding and genetics of crops with complex genomes.

## Introduction

Polyploid species contain more than two sets of chromosomes (Comai, 2005). It is estimated that polyploid species account for up to 80% of living plants (Otto, 2007; Rieseberg and Willis, 2007). The occurrence of polyploidy can be explained by two major mechanisms: (1) the failure of meiosis or mitosis, and the fusion of unreduced gametes (Comai, 2005), which results in genome doubling in a single cell and produces autopolyploid species, such as cultivated potato (Watanabe, 2015); and (2) the combination of two or more genomes from different but related species through hybridization and subsequent chromosome doubling, which produces allopolyploid species, such as bread wheat (Chen and Ni, 2006; Marcussen et al., 2014; Borrill et al., 2015). Polyploidization has become a common approach to overcome the sterility of interspecific hybrids during plant breeding. For example, triticale is a hybrid derived from the cross between wheat (*Triticum turgidum*) and rye (*Secale cereal*) (Gupta and Priyadarshan, 1982). After polyploidization, triticale is able to be propagated from seed, and is now widely grown as a forage in 37 countries across the world (http://www.fao.org/faostat/en/#data/QC). Based on time of origin, polyploids can be classified as either neopolyploids or paleopolyploids. Neopolyploids are any newly formed polyploid without diploidized genomes, such as potato, cotton, canola, wheat, and sugarcane. On the other hand, paleopolyploids are formed at least several million years ago with ancient and diploidized genomes, such as maize and soybean (Hilu, 1993; Tayalé and Parisod, 2013). The chromosome numbers of gametes of neopolyploids are usually multiples of the basic chromosome number in the genera (Hilu, 1993), while paleopolyploids usually have large basic chromosome numbers (mostly larger than *n* = 13) (Guerra, 2008).

Polyploidy is an important force during plant evolution. The duplication or addition of the genomes involves molecular and physiological adjustments in plants, such as changing gene expressions, and generating novel phenotypes (Adams and Wendel, 2005). Moreover, new species can be generated due to polyploidization (Soltis et al., 2015). It was estimated that 15 and 31% of speciation events in angiosperms and ferns, respectively, are involved in ploidy changes (Wood et al., 2009). Different phenotypic consequences were observed for polyploids. For example, in allopolyploid wheat, there are various phenotypic consequences of polyploidy depending on the relationship of homoeologs, such as accelerated senescence (dosage effects), changing growth habit (homoeolog dominance), and changing specific domestication traits (interaction of homoeologs) (Borrill et al., 2015). The combination of two or more sets of chromosomes from different species in allopolyploids through hybridization can lead to heterosis (Chen, 2013). This may be explained by the allelic interactions or epigenetic modifications of some key genes. By contrast, autopolyploids, in comparison with their respective diploids, experience neither a significant genome restructuring nor a strong alteration of gene expressions (Parisod et al., 2010). However, A few genes whose expressions and final phenotypes, such as cell size and organ thickness, are dramatically changed and linearly correlated with the ploidy (Stupar et al., 2007). The role of polyploidy in plant evolution has been reviewed elsewhere (Adams and Wendel, 2005; Madlung, 2013; Soltis et al., 2015), and thus not discussed in details in this review.

Despite of the popularity and importance of polyploid species in agriculture, in the genomic era, improvement and molecular breeding of polyploid crops has lagged behind many diploid crop species largely due to the complexity of the genome composition. As a result, polyploid genetic studies have also fallen behind. Over the past decade, crop genetic studies and molecular breeding particularly for diploid crops, have achieved remarkable success owing to the application of molecular marker technologies (Würschum et al., 2013; García-Pereira et al., 2014; Ergül et al., 2015). With the advantages of abundance, cost-efficiency, and high-throughput assays, single nucleotide polymorphism (SNP) has become increasingly important in crop genetic studies (Seeb et al., 2011), such as association mapping and genomic selection (Cavanagh et al., 2013; Huang and Han, 2014; Allwright and Taylor, 2016).

As large amounts of SNPs have been discovered, the demand of high-throughput SNP genotyping has increased. SNP genotyping technologies include the low-throughput gel-based approach, the cleaved amplified polymorphic sequence (CAPS) marker approach (Thiel et al., 2004), PCR-based fluorescently-labeled high-throughput methods, high-resolution melting (HRM) curve analysis, TaqMan® and KASP™ assay (Martino et al., 2010), fixed array systems such as Illumina Infinuium (Mason et al., 2017), Affymetrix Axiom (Allen et al., 2017), and next generation sequencing (NGS) enabled approaches such as restriction-enzyme-based genotyping by sequencing (GBS) (Thomson, 2014). The time, flexibility, and cost-effectiveness of the above SNP genotyping technologies has been well-discussed by Thomson (2014).

Currently, the most popular high throughput genotyping platforms are the hybridization based SNP array and various NGS enabled genotyping such as GBS, which refers to any platform that uses sequencing to obtain genotypes and a total of 13 different GBS methods have been summarized (Scheben et al., 2017). GBS has been successfully utilized in crop species mainly due to the rapid developments of sequencing technologies, increasing read length, and more available reference genomes. The utilization of GBS in plants as well as its advantages and disadvantages have been well-summarized and reviewed (Deschamps et al., 2012; He et al., 2014; Rasheed et al., 2017). SNP array is a type of DNA microarray containing designed probes harboring the SNP positions, which is hybridized with fragmented DNA to determine the specific alleles of all SNPs on the array for the hybridized DNA sample (LaFramboise, 2009). Many SNP arrays have been successfully applied in diploid species genotyping, such as the Apple 480K SNP array (Bianco et al., 2016), the Maize 600K SNP array (Unterseer et al., 2014), and the Rice 700K SNP array (McCouch et al., 2016). However, SNP identification and utilization for genotyping in polyploid species has progressed slowly due to the complex nature of genomes and inheritance of polyploids. Although SNP identification in highly polyploid species has more obstacles than in diploids as reviewed by Clevenger et al. (2015), the development of SNP arrays in polyploids has achieved noticeable progress (Bassil et al., 2015; Aitken et al., 2016; Clevenger et al., 2017).

While SNP array and GBS have their own pros and cons, they could complement each other. To date, SNP identification in polyploids (Kaur et al., 2012; Clevenger et al., 2015) and SNP array development mainly in diploid species (Rasheed et al., 2017) have been extensively reviewed. However, the summarization and discussion of SNP array in polyploid crop species is lacking. The review from Kaur et al. (2012) mainly focused on SNP identification and validation in allopolyploids. Little attention was paid to autopolyploids or genotyping using SNP array technology. As the development of SNP array for polyploid crops is more complicated than diploids, it would be helpful to review the current progress of SNP array development and applications in polyploid crops. The current review focuses on following aspects in both autopolyploid and allopolyploid crops: (1) comparing SNP array technology with other methods, especially NGS-based technologies for genotyping polyploids; (2) explaining SNP selection criteria for array design for polyploids; (3) summarizing currently available arrays and their applications in polyploid crops; (4) discussing available SNP array genotype calling software; and (5) providing suggestions and future perspectives of SNP array application in polyploid crops.

## SNP identification in polyploids

In this section we briefly describe how the SNPs are identified in polyploid species. The first step for SNP array development is SNP identification from the DNA or dDNA sequences. Over the past decade, the cost and running time for NGS technologies have dramatically reduced, thus they have been extensively used for genome and transcriptome sequencing for a wide range of species including polyploids. The substantial sequences generated from the NGS served as a valuable reservoir for SNP identification. Several NGS enabled approaches were also developed specifically for high throughput genotyping such as GBS, exome sequencing (Exom-seq), restriction site associated DNA sequencing (RAD-seq), and the double-digest RAD-seq (ddRAD-seq) (Peterson et al., 2012; Borrill et al., 2015). Therefore, these NGS enabled genotyping methods have identified a substantial number of SNPs in many crop species including polyploid crops. For example, as the most widely used NGS-enabled genotyping method, GBS (Elshire et al., 2011) was reported in identifying and assaying SNPs in several polyploids: 20K SNPs from 164 wheat DH lines (Poland et al., 2012), 76K SNPs from 14 sugarcane accessions (Yang et al., 2017), and 84K SNPs from 151 sugarcane clones (Balsalobre et al., 2017).

SNP identification pipelines based on NGS data generally include NGS reads mapping or alignment, SNP calling, filtering, and validation, which have been reviewed for polyploids (Clevenger et al., 2015). One notable challenge of using these NGS enabled approaches for genotyping polyploid species is that the required read depth is much higher than that of diploid species. For example, diploids require 4~6x read depth for sequencing large number samples (around 400) (Le and Durbin, 2011) or 7.7x depth for sequencing 99% of the alleles (Clevenger et al., 2015); while the suggested depth for polyploids was up to 48.7x in octoploid strawberry (Bassil et al., 2015), 60x in tetraploid potato (Hamilton et al., 2011), and 100x in sugarcane (Song et al., 2016), which would require a high depth of sequencing (Hamilton et al., 2011; Wang et al., 2014; Bassil et al., 2015; Aitken et al., 2016; Clevenger et al., 2017). The depth can be even higher if different SNP dosages need to be called. For allopolyploids, distinguishing homologous SNPs between genotypes from homoeologous SNPs between subgenomes or paralogous SNPs between duplicated gene copies is a daunting task due to the high sequence similarity between the subgenomes (Kaur et al., 2012; Dufresne et al., 2014; Clevenger et al., 2015). Take peanut (AABB genome) as an example, the two sub-genomes of peanut are highly similar (96% median identity; Bertioli et al., 2016), thus the homoeologous SNPs account for a large proportion of identified SNPs (Clevenger et al., 2015; Peng et al., 2016). While homoeologous variations may hinder genomic analyses, the understanding of homoeologs can be helpful for adjusting the response of quantitative traits in polyploid crops. Since the functional redundancy of homoeologs can “lock up” some phenotypic variation, as was reported in wheat, the manipulation of homoeologs will likely unlock the full polyploidy potential of the crop (Borrill et al., 2015). The factors influencing the discrimination of these different types of SNP classes and the status of SNP discovery in allopolyploid crops have been reviewed elsewhere (Kaur et al., 2012; Clevenger et al., 2015).

Beside the NGS enabled genotyping approaches, datasets of published DNA sequences or expressed sequence tags (ESTs) also are valuable sources for SNP discovery in polyploids. For example, 8,327 SNP were identified from the Sanger EST datasets of three potato cultivars and finally filtered to 2,358 high quality SNPs by Hamilton et al. (2011). Similarly, Tinker et al. discovered 7,680 SNPs from four genomic DNA sequence sources in NCBI, which were derived from more than 20 diverse oat cultivars (Tinker et al., 2014). In practice, selection of SNP discovery technology may largely rely on the availability of SNP resources, and the specific biological question of interest.

## Features of SNP array technology

SNP array is a high-throughput, relatively cost-efficient, and automatically genotyping assay. It has been widely used in genetic studies of crops, including genome-wide association studies (GWAS) (Chen et al., 2014; McCouch et al., 2016), linkage map construction (Ganal et al., 2011; Felcher et al., 2012), genomic selection (Yu et al., 2014; Clarke et al., 2016), population structure analysis (Unterseer et al., 2014; Hulse-Kemp et al., 2015; Wang et al., 2016), and gene mapping (Dalton-Morgan et al., 2014). The capacity of currently available SNP arrays is up to 700K in diploids (rice) (McCouch et al., 2016), 487K in triploids (apple) (Bianco et al., 2016), 58K in tetraploids (peanut) (Clevenger et al., 2017; Pandey et al., 2017), 820K in hexaploids (wheat) (Winfield et al., 2016), 90K in octoploids (strawberry) (Bassil et al., 2015), and 345K in dodecaploids (sugarcane) (Aitken et al., 2016).

SNP array technology, similar to many other biotechnologies, has its pros and cons. For high-throughput genotyping, SNP array has several advantages over other genotyping approaches. First, SNP array data is relatively easy to analyze compared to data generated using NGS-based methods. Particularly when considering labor-intensive NGS library preparation (Garvin et al., 2010) and downstream bioinformatics data analysis investment for accurate SNP calling (Liu et al., 2012; Torkamaneh et al., 2017). To be more specific, the genotypes of SNP markers can be called and provided by Affymetrix or Illumina, or researchers also can call genotypes following the Affymetrix or Illumina genotype calling pipeline according to the user guide. However, it is more difficult and time-consuming to call genotypes using NGS-based data in polyploid species. As mentioned above, SNP genotype calling includes reads trimming, reads alignment, SNP calling, SNP filtering, etc. (Clevenger et al., 2015), which requires the background of bioinformatics. Second, SNPs from genomic regions of interest can be specifically included on the array. In addition, the number of interested SNPs to be placed on the array is flexible for Illumina and Affymetrix platforms. Third, SNP array is considered as low to moderate costs of per sample, despite the significant decrease in costs associated with NGS. It costs $28~$90 (USD) per sample for Affymetrix Axiom SNP array (personal communication), while NGS approaches have the lowest price for GBS at $35 per sample, followed by RNA-seq at $260 per sample, and whole genome sequencing (WGS) at >$500 per sample (Peng et al., 2017). As polyploid crops usually have a large genome size, the NGS-based methods would require a huge amount of sequencing data for adequate coverage.

SNP array and NGS can complement each other. Though NGS has absolute advantages in generating sequence and identification of variants, its genotyping in polyploids is still hindered. SNP array is still a good option as a genotyping platform based on the increasing variants discovered by NGS methods. However, SNP array has its own shortcomings, such as required prior genomic information, only genotyping known SNP locations, and manual dosage scoring (in some case) (Wang et al., 2014; Vos et al., 2015). In addition, its design and further optimization can be time consuming. Ascertainment bias is a common issue for genotyping arrays, which is due to non-random sampling of polymorphisms in the population of interest (Heslot et al., 2013) or due to a small number of samples used as SNP discovery panels (Albrechtsen et al., 2010). For example, a small sample size may mainly capture common alleles, excluding rare alleles (Gravel et al., 2011). This would likely distort subsequent genetic inferences. Efforts have been taken to reduce ascertainment bias such as adopting whole genome sequencing with high coverage, updating the markers on the SNP array, and combining markers from multiple arrays.

The comparison of SNP validation rate between SNP array and NGS-based methods would be difficult, since factors like population type (bi-parental or natural population) and population size used for validation will have a great impact on the validation rate. For example, when genotyping a total of 108 hexaploid wheat varieties, a 900K SNP array in wheat showed 99.8K polymorphic SNP probes. However, when genotyping a total of 475 accessions including the relatives of those varieties and progenitors, the number of polymorphic probes increased to 453.1K (Winfield et al., 2016). Thus, only the validation rate of SNP array has been summarized and discussed as follows. Currently, SNP array has been applied in many polyploid crops and most of the arrays achieved decent polymorphic rates, as summarized in Table 1. Among the 16 SNP arrays in nine polyploid crop species, eight (50%) SNP arrays had a polymorphic rate ≥80%, and four (25%) had a polymorphic rate between 60 and 70%. Only two SNP arrays had a polymorphic rate of less than 60%, to be specific, 42.7% in oat (Oliver et al., 2013), and 56.8% in sugarcane (Aitken et al., 2016).

**Table 1:** Summary of SNP arrays developed in polyploid crops.

**Species**	**SNP source**	**Array size**	**Genotyping sample size**	**SNP efficiency**	**Application**	**Reference**
Peanut (allo-tetraploid, 2*n* = 4x = 40)	163K SNPs from DNA re-sequencing of 38 accessions and RNA-sequencing of 3 accessions	58K (58,233) Axiom (Affymetrix)	297 accessions from 48 countries, including 36 wide species	44,424 (73.3%) polymorphism	Genetic diversity and genetic architecture	Pandey et al., 2017
Potato tetraploid (auto-tetraploid, 2*n* = 4x = 48)	Two million SNPs from RNA-seq of 3 commercial cultivars and 8K SNPs from Sanger EST of 3 additional cultivars	96 Illumina BeadXpress (Illumina)	248 lines	82 (85.4%) reliably scored	Genetic diversity analysis (population structure)	Hamilton et al., 2011
Potato tetraploid (auto-tetraploid, 2*n* = 4x = 48)	69K high confidence SNPs from previous identification (Hamilton et al., 2011)	8K (8,303) Infinium (Illumina)	184 progeny, 92 from population D84 and 92 from population DRH	Over 4,400 (53.0%) markers were mapped	Development of linkage maps	Felcher et al., 2012
Potato tetraploid (auto-tetraploid, 2*n* = 4x = 48)	20K SNPs from previous identification (Hamilton et al., 2011; Uitdewilligen et al., 2013)	18K (17,987) Infinium (Illumina)	569 accessions, including 537 tetraploids and 32 diploids	14,530 (80.8%) successfully scored with fitTetra	Reconstruction of the breeding history, shaping the genetic composition	Vos et al., 2015
Cotton (allo-tetraploid, 2*n* = 4x = 52)	50K SNPs from 9 intra-specific data sets, and 20K SNPs from 4 inter-specific data sets (11 previous studies and 2 unpublished studies)	63K (63,058) Infinium (Illumina)	1,156 individual samples	38,822 (61.6%) polymorphic markers	Development of high density genetic map	Hulse-Kemp et al., 2015
Alfalfa (auto-tetraploid, 2*n* = 4x = 32)	900K SNPs from RNA-sequence of 27 alfalfa genotypes (including 23 tetraploid and 4 diploid) by previous reported (Li et al., 2012)	9K (9,277) Infinium (Illumina)	280 diverse genotypes including related species	7,476 (81%) polymorphic markers	Evaluation population structure and linkage disequilibrium	Li et al., 2014b
Brassica (allo-tetraploid, 2*n* = 4x = 38)	54K SNPs identified and evaluated by previous reports (Bus et al., 2012 and Dalton-Morgan et al., 2014) and unpublished data; 24M SNPs discovered from published sequence data sets (Harper et al., 2012 and Clarke et al., 2013)	52K (52,157) Infinium (Illumina)	437 diverse genotypes (432 diverse genotypes were generated independently in two laboratories)	About 60% genome-specific markers	Genetic map generation	Clarke et al., 2016
Wheat (allo-hexaploid, 2*n* = 6x = 42)	25K SNPs from RNA-sequence of 26 hexaploid accessions	9K (9,000) Infinium (Illumina)	2,994 hexaploid accessions, including landraces and modern cultivars	7,733 (85.9%) successfully genotyped	Genetic diversity and population structure, selection scans	Cavanagh et al., 2013
Wheat (allo-hexaploid, 2*n* = 6x = 42)	128K SNPs from RNA-sequencing of 19 hexaploid and 18 tetraploid accessions	90K (91,829) Infinium (Illumina)	646 accesions, including 55 tetraploid cultivars, 447 hexaploid cultivars, and 144 hexaploid landraces	81,587 (89%) produced functional assays	Characterization of genomic diversity	Wang et al., 2014
Wheat (allo-hexaploid, 2*n* = 6x = 42)	921K SNPs from Exom sequencing of 14 diploid, 5 tetraploid, 23 hexaploid, and 1 decaploid accessions	820K (819,571) Axiom (Affymetrix)	475 accessions, including diploid, tetraploid, and hexaploid wheat accessions and wheat relatives	546,299 (66.7%) polymorphic SNPs	Physical and genetic mapping, genetic characterization	Winfield et al., 2016
Wheat (allo-hexaploid, 2*n* = 6x = 42)	35K SNPs from previous study (Winfield et al., 2016)	35K (35,143) Axiom (Affymetrix)	1,843 DNA samples, including 1,779 unique hexaploid wheat accessions and 64 replicates	33,326 (94.8%) polymorphic SNPs	Genetic mapping, and characterization of genetic diversity	Allen et al., 2017
Oat (allo-hexaploid, 2*n* = 6x = 42)	11K high-confidence SNPs from RNA-sequencing of 20 genotypes (Oliver et al., 2011)	3K (3,072) GoldenGate (Illumina)	390 recombinant inbred lines	1,311 (42.7%, success rate) robust markers	Development of physically anchored consensus map	Oliver et al., 2013
Oat (allo-hexaploid, 2*n* = 6x = 42)	8K SNPs from 4 DNA sequence data sets. (Tinker et al., 2009; Poland et al., 2012; Oliver et al., 2013)	6K (5,743) Infinium (Illumina)	1,110 hexaploid samples, including 109 diverse cultivars, 390 progeny, and 595 breeding lines	4,975 (86.6%) SNPs produced successfully assays	SNP discovery and annotation, population genetic characteristics	Tinker et al., 2014
Strawberry (allo-octploid, 2*n* = 8x = 56)	160K di-allelic SNPs from DNA-sequencing of 19 octoploid and 6 diploid strawberry accessions	90K (95,062) Axiom (Affymetrix)	384 samples, including 357 octoploid accessions and cultivars, 4 diploid accessions, and 23 progeny.	60,473 (64%) polymorphic SNPs, including 23,355 (24.6%, success rate) markers in *PHR*	High density linkage maps, QTL identification	Bassil et al., 2015
Sugarcane (auto-dodecaploid, 2*n* = 12x = 100~120)	2.6M SNPs from target gene-rich regions sequencing of 16 lines	345K (345,704) Axiom (Affymetrix)	367 clones, including parental clones, cultivars, and unselected families	48,802 (14.1%) validated polymorphic markers and 11,443 (3.3%, success rate) markers in *PHR*	Association analysis of cane yield and sugar content, genetic mapping	Aitken et al., 2016

*PHR, Poly High Resolution, which were polymorphic and passed all quality control (Bassil et al., 2015)*.

## SNP selection for SNP array design and genotype calling in polyploid species

Technically, two platforms have been used for SNP array in polyploid species, Illumina (Dalton-Morgan et al., 2014; Vos et al., 2015; Clarke et al., 2016) and Affymetrix (Bassil et al., 2015; Clevenger et al., 2017; Pandey et al., 2017). Different chemistries and computational algorithms are used for assay and genotype calling between Affymetrix and Illumina SNP arrays, the hybridization principle (complementary base pairing) and captured signal intensity principle (calculation of amount of target DNA and the affinity between target DNA and probes) are alike (LaFramboise, 2009).

For SNP marker selection in development of the array, most of the criteria are the same between Illumina and Affymetrix, except for two parameters: (1) sequence length on either side of the SNP (50 bp for Illumina vs. 20 bp for Affymetrix), and (2) evaluation parameters (Illumina Assay Design Tool (ADT) value vs. Affymetrix P-convert value). The general considerations for array SNP selection include SNP depth, SNP types, SNP frequency, additional variations within probe sequence of target SNPs, and probe sequence parameters. Specifically, (1) The average SNP read depth, or single genotype SNP depth is considered as it is related to the accuracy of SNPs called. If the depth is too low, the SNPs could be called due to sequence errors. If the depth is too high, the SNPs may be called from repetitive sequences. Thus, a range of depths for different crop species is recommended (Deulvot et al., 2010; Chagné et al., 2012; Bianco et al., 2014). (2) There are two types of SNPs: transition SNPs such as A/G, T/C, and transversion SNPs such as A/T, C/G, A/C, and T/G. For SNP array development, the transition SNP type is preferred and transversion SNPs, INDELs, or multiple allelic SNPs are typically excluded (Bianco et al., 2016; Clarke et al., 2016). Particularly, A/T or C/G SNPs are eliminated, as these types require two probes, while other SNP types require just one probe for genotyping (Dalton-Morgan et al., 2014; Clevenger et al., 2017). (3) SNPs with very rare alleles (present in less than two genotypes), which most likely could be due to sequence or alignment errors, are eliminated from the array (Clarke et al., 2016). (4) Additional variants within the probe sequence is a definite consideration for exclusion. The Affymetrix Axiom technology is more tolerant to any additional variants present in the probe sequence than Illumina Infinium, since Affymetrix removes SNPs (labeled as not recommended) with additional variants within 20 bp up or downstream of target SNPs (Bassil et al., 2015), while it's 50 bp for Illumina (Dalton-Morgan et al., 2014; Clarke et al., 2016). (5) Before probe design for the SNP array, the flanking sequences of the target SNPs are thoroughly evaluated by Affymetrix P-convert value or Illumina ADT value. The P-convert value predicts the probability of SNP converting on the array, which is used to assign forward or reverse probes for each SNP by considering probe sequence, binding energies, impacts from adjacent SNPs, etc. (Liu et al., 2014; Qi et al., 2017). Similarly, the ADT value predicts a likelihood of success for requested SNP loci (https://www.illumina.com/literature.html). SNPs are kept if the Affymetrix P-convert value or Illumina ADT value is higher than 0.6 for both forward and reverse probes. Sometimes, SNPs distribution, such as spreading over all chromosomes evenly or in exonic region (Deulvot et al., 2010; Chagné et al., 2012) are taken into consideration. For allopolyploids, the chosen SNPs should be evenly distributed across the sub-genomes. In addition, genome-specific SNPs should be included, since their segregation patterns follow that of a diploid, as in peanut (Clevenger et al., 2017) and wheat (Cavanagh et al., 2013). Moreover, inter-specific SNPs can be included to reduce ascertainment bias for array design, which has been done in cotton (Hulse-Kemp et al., 2015).

Several different possible dosages are available for a specific SNP in polyploids, which should also be taken into account when selecting SNPs. This is specifically an issue for autopolyploids with a high ploidy, such as sugarcane. The estimation of SNP dosages during SNP mining stage has been reported in sugarcane. In a study aiming at SNP discovery in sugarcane, the different dosage levels of SNPs were called by using UnifiedGenotyper in the Genome Analysis ToolKit (GATK) (Song et al., 2016). Since gene dosage and allelic configuration are unknown, there is a paucity of statistical approaches (Luo et al., 2001; Baker et al., 2010) for linkage analysis using all the markers with different dosages. Therefore, single dose markers (SDMs) (Wu et al., 1992) have become the primary marker choice for linkage analysis in polyploids like sugarcane, whose segregation pattern follows that of a diploid (1:1 and 3:1 in F1 populations or 3:1 in selfing populations) (Pastina et al., 2012; Vukosavljev et al., 2016; Balsalobre et al., 2017). This makes many available statistical methods for diploids applicable for polyploids (Baker et al., 2010). The proportion of SDMs in the markers generated from high throughput NGS data can be high. In a study mining sequence variations among sugarcane accessions, the percentage of single dose SNPs ranged from 38.3 to 62.3% with an averaging of 49.6% (Yang et al., 2017). Another research group developing a 345K sugarcane SNP array specifically included single dose SNPs as much as possible (Aitken et al., 2016).

Above are the general and technical considerations in SNP selection process for Illumina and Affymetrix platforms. In certain cases, if there are multiple sources of SNPs called from different sequence approaches, then SNP source should be taken into account. It was reported in polyploid species that SNP validation rates of SNP array were associated with the SNP identification approaches (Hulse-Kemp et al., 2015). SNPs derived from gene-enriched sequencing had a higher marker validation rate (88%), such as RNA-seq and gene-enrichment restriction libraries from five *G. hirsutum* lines, than SNPs from genomic re-sequencing (50%) from 12 *G. hirsutum* lines (including the five lines from RNA-seq). Similar validation rates were obtained using RNA-seq in *Eucalyptus grandis* (83%) (Novaes et al., 2008) and *Brassica napus* (87%) (Barbazuk et al., 2007). Therefore, RNA-seq and gene-enrichment are common strategies to identify SNPs for SNP array development in polyloids including peanut (Hulse-Kemp et al., 2015), potato (Hamilton et al., 2011), wheat (Cavanagh et al., 2013; Wang et al., 2014), and sugarcane (Aitken et al., 2016).

## SNP array development and applications in polyploid species

Although complicated, several recent studies have reported the development and application of SNP array in polyploid crops (Table 1) including tetraploid, hexaploid, octoploid, and even dodecaploid species.

### Tetraploid

The recent progress in SNP array development in tetraploid species was made in the allotetraploid peanut (*Arachis hypogaea*; AABB-type genome; 2*n* = 4x = 40; ~2.7 Gb genome size) (Bertioli et al., 2016). A total of 163.8K SNPs were identified from 30 represented tetraploid cultivars and 11 wild diploid accessions by DNA resequencing and RNA-seq methods, of which 58K SNPs were selected for developing a peanut SNP array (Pandey et al., 2017). The array was further utilized to investigate the genetic architecture of 300 diverse accessions, which showed 44,424 (73.3%) polymorphic SNPs on this array (Pandey et al., 2017). Meanwhile, the validation of the 58K SNP array among 384 diverse cultivars revealed 54,564 (93.7%) polymorphic SNPs between diploid species, 47,116 (81.0%) polymorphic SNPs between cultivars and interspecific hybrids, and 15,897 (27.3%) polymorphic SNPs within *A. hypogaea* accessions (Clevenger et al., 2017).

Potato (*Solanum tuberosum* L.; 2*n* = 4x = 48; ~844 Mb genome size) is a highly heterozygous autotetraploid, and is the most important non-grain food crop in the world (Consortium, 2011). Three SNP arrays were developed for this crop, 96 BeadXpress SNP Array (Hamilton et al., 2011), 8K Infinium Potato Array (Felcher et al., 2012), and 20K Infinium SNP Array (Vos et al., 2015). Through transcriptome sequencing and Sanger EST sequencing, a total of 69,011 high confidence SNPs were identified by Hamilton et al. (2011), of which 96 SNPs were selected for the 96 BeadXpress SNP array development. This study revealed distinct relationships among different potato market classes. In addition, another 8,303 SNPs were selected from the same SNP set for 8K Infinium Potato Array development for linkage mapping (Felcher et al., 2012). By using previously identified SNPs from two studies (Hamilton et al., 2011; Uitdewilligen et al., 2013), the 20K SNP array was designed, which was performed on 569 potato accessions to study drift and selection effects that influenced the genetic components of European potato. The array was also deployed to evaluate and estimate linkage disequilibrium (LD) decay in another study (Vos et al., 2017). In addition, this 20K SNP array was used to evaluate the double-reduction (DR) landscape in 237 individuals, which showed the phenomenon that the rate of DR increased with the distance from the centromeres (Bourke et al., 2015). Cotton (*Gossypium hirsutum* L.; 2*n* = 4x = 52; ~2.5 Gb genome size) is widely cultivated, providing over 95% of cotton production in the world (Zhang et al., 2008). A cotton 63K SNP array was developed based on identified SNPs from 13 different data sets, thus covering a diversity range of SNP sources, containing 45K intra-specific SNPs and 18K inter-specific SNPs. This array was applied successfully to distinguish differences between *G.hirustum* and other Gossypium species, between wild and cultivated genotypes, and among cultivars (Hinze et al., 2017). It was also used to produce two high-density genetic maps (Hulse-Kemp et al., 2015), and to identify 160 quantitative trait loci (QTLs) related to 16 agronomic traits by GWAS among 503 *G.hirustum* accessions (Huang et al., 2017).

Alfalfa (*Medicago sativa* L.; 2*n* = 4x = 32) is the main forage legume crop in the world (Li et al., 2014b). It plays important ecological roles in livestock farming systems by stabilizing soil and increasing soil fertility through symbiotic biological nitrogen fixation (Li et al., 2014b; De Vega et al., 2015; O'Rourke et al., 2015). A total of 900K SNPs were identified between 27 diverse alfalfa genotypes by RNA-seq (Li et al., 2012), of which 9K SNPs were used to develop an Illumina alfalfa SNP array (Li et al., 2014a). To validate this SNP array, 280 alfalfa genotypes were assayed with an 81% SNP marker polymorphic rate, and results showed clear population structure, analyzed genetic diversity of sub-populations, and evaluated the LD across all genotypes (Li et al., 2014a).

Oilseed rape (*Brassica napus* L., AACC type-genome; 2*n* = 4x = 38; ~845 Mb genome size) is an essential economical oilseed crop, which can be used for extracting oil from seed and edible vegetable (Chalhoub et al., 2014; Clarke et al., 2016). A 60K Brassica Infinium array was designed by Clarke et al. (2016). The sources of SNPs used on this array mainly included previously identified SNPs (Bus et al., 2012; Dalton-Morgan et al., 2014; Cheung et al. unpublished) and newly called SNPs in this study originating from published sequence data in Harper et al. (2012), Clarke et al. (2013) and unpublished genomic and transcriptome sequence data in Clarke et al. To validate this 60K array, 327 and 432 diverse genotypes were independently genotyped at two laboratories, which obtained ~60% of genome specific markers in diverse *B. napus* genotypes, 26.5 and 29.7K scorable SNP markers in *B. oleracea* and *B. rapa* respectively, and a map of *B. napus* with 46% (21,766 SNPs) mapped markers in one of DH population was constructed (Clarke et al., 2016). This 60K *Brassica* SNP array has been widely used for oilseed rape, such as identification of three QTLs associated with Sclerotinia stem rot resistance (Wu et al., 2016), 79 QTLs related with seed quality, flowering time, and root morphology traits (Wang et al., 2017), 117 genomic regions involved in selective sweeps (Zou et al., 2018), etc. In addition, a user guide was reported for this Brassica 60K SNP array (Mason et al., 2017).

### Hexaploid

Wheat (*Triticum aestivnm* L.; AABBDD type-genome; 2*n* = 6x = 42) is one of the world's most important cereal crops and has the most progresses in SNP array development among hexaploid crop species (Consortium, 2014). A 9K iSelect SNP array was used to assess genetic variations in coding regions of 2,994 hexaploid wheat accessions (Cavanagh et al., 2013), and a high density SNP map was constructed. Subsequently, a 90K SNP iSelect array was developed and used to assess genetic variations in allohexaploid and allotetraploid wheat populations (Wang et al., 2014). With the 90K SNP iSelect array, researchers working on wheat were able to detect QTLs conveying leaf rust resistance (Gao L. et al., 2016), identify QTLs associated with physiological and agronomic traits (Gao F. et al., 2016; Zou et al., 2016), perform phylogenetic analysis (Turuspekov et al., 2015), and detect candidate loci involved in domestication and improvement (Gao et al., 2017). Recently, Winfield et al. (2016) used Exom-seq to identify 921K putative SNPs from 43 bread wheat accessions. A total of 820K SNPs were included for SNP array, which were then validated on 475 accessions with an average call rate of 98.4%. This array was also used to map three populations and characterize wheat accessions and relatives. Furthermore, to update this 820K SNP array, with consideration of higher level polymorphic and more evenly distributed SNPs, 35K SNPs were selected to develop a commercial high-density Axiom array. This 35K SNP array was used to assess a diverse panel of 1,843 samples, which constructed genetic maps and characterized novel genetic diversity among those samples (Allen et al., 2017).

Cultivated hexaploid oat (*Avena sativa* L.; AACCDD type-genome; 2*n* = 6x = 42) is one of the important cereal crops in the world (Andon and Anderson, 2008). The first oat SNP array contained 3,072 SNPs, which was applied to build a physically anchored consensus map of oat with 985 mapped SNPs (Oliver et al., 2013). To expand this SNP array, various bioinformatic pipelines were applied to discover SNPs from multiple available DNA sequence sources, including eight alternate methods, and a new SNP calling method was also assessed by Tinker et al. (2014). Finally, a 6K oat BeadChip was designed, which produced 86.6% success rate by validating in 1,100 samples (Tinker et al., 2014). Recently, this 6K SNP array was applied to genotype 138 oat accessions for mapping QTL associated with frost tolerance using GWAS (Tumino et al., 2016).

### Octoploid and dodecaploid

Cultivated strawberry [*Fragaria* × *ananassa* (Duch.); AABBCCDD genome; 2*n* = 8x = 56, ~698 Mb genome size] is an allo-octoploid crop species (Hirakawa et al., 2014), while *Fragaria* (2*n* = 2x = 14, genome size of 240 Mb) is a diploid, called woodland strawberry (Shulaev et al., 2011). A total of 36 million unique variants were identified as SNP resources among 19 octoploid and six diploid strawberry accessions, from which a 90K SNP array (ISTRAW90) was designed (Bassil et al., 2015). The ISTRAW90 array was validated by genotyping 384 octoploid strawberry samples, and 12,609 SNPs had the highest quality, which exhibited relatively low success rate (13.3%). However, it is still a useful high-throughput tool to construct high density linkage maps (Mahoney et al., 2016), to distinguish cultivars [application of Poly High Resolution *(PHR)* SNPs] (Jung et al., 2017), and to perform genomic selection (Gezan et al., 2017) in octoploid strawberry.

As a highly heterozygous species, commercial sugarcane (*Saccharum* complex, 2*n* = 12x = 100~120, ~10 Gb genome size), is autopolyploid mostly derived from the interspecific cross between auto-octoploid *S. officinarum* (2*n* = 8x = 80) and autoploid *S. spontaneum* (2*n* = 4x–16x = 32~128) (Roach, 1989; D'hont et al., 1998) followed by backcrosses with *S. officinarum*. Sugarcane is not only highly heterozygous, but also exhibits chromosome number variation, mixed ploidy, as well as aneuploidy. To identify large numbers of SNPs for development of sugarcane SNP array, 16 sugarcane clones were used for deep sequencing with target on gene-rich regions, which identified 4.5M SNPs (Aitken et al., 2016). Based on the combination of different allele dosage levels, the 4.5M SNPs were clustered into three classes and evaluated by Affymetrix. Finally, a total of 345K SNPs were selected with high quality score from all classes and with wide distribution across sugarcane contigs. Subsequently, a 345K sugarcane SNP array was developed and used for genotyping 367 sugarcane clones, resulting in 48,802 (14.1%) validated polymorphic markers for further analysis and 11,443 (3.3%) highest quality markers. These 48,802 polymorphic markers have been included in a 50K cost-effective SNP array to genotype over 2,000 sugarcane clones in Australia (Aitken et al., 2016).

## SNP array genotype calling and data analysis pipelines

After the SNP array assay, calling SNP genotypes from the assay is a critical next step. For polyploid species, the genotyping calling is complicated. Rather than having three possible genotypes (homozygote with reference allele, heterozygote, and homozygote with alternative allele) at a SNP locus in diploid species, polyploid species usually have more than three possible genotypes. Theoretically, the number of genotypes can be up to five in tetraploids, seven in hexaploids, nine in octoploids, and 13 in dodecaploid. So far, there are two software, fitTetra and ClusterCall, written for tetraploids, which can call five genotypes. Another software, SuperMASSA, was written for all ploidies (so far only successfully reported in sugarcane). Most other polyploid crops were using genotype calling software accompanying Affymetrix or Illumina platform. However, the disadvantage of the software is their inability to identify >3 clusters for Affymetrix or >5 clusters for Illumina platform. The GenomeStudio software from Illumina is able to provide five clusters. As an example, the markers from the 20K Infinium SNP Array (Vos et al., 2015) were first automatically scored using fitTetra, after which the rejected markers were further manually scored using GenomeStudio. A total of 843 markers were recovered from the 1,832 rejected markers with a 46% recovery rate (Vos et al., 2015). Consequently, the GenomeStudio may be impractical to use for large amounts of markers, as it requires manual adjustment of the cluster boundaries for each marker.

There are different scenarios for genotyping calling for autopolyploids and allopolyploids. The difficulty of SNP calling in allopolyploids mainly comes from the complexity of genome architecture. Ideally, the allelic SNP variation should be derived for each sub-genome, which is why sub-genome specific SNPs are desired (Kaur et al., 2012). With sub-genome specific SNPs, the genotype calling in allopolyploids becomes no different with diploids. For example, in an Affymetrix Axiom SNP array of strawberry and an Illumina Infinium SNP array of cotton, the sub-genome specific SNPs would produce three distinct clusters, behaving like a co-dominant marker in diploids with two homozygous genotypes and one heterozygous genotype (Bassil et al., 2015; Hulse-Kemp et al., 2015). These types of SNPs are distinct from the homoeologous SNPs that are polymorphic between sub-genomes within a sample. In practice, the sub-genome specific SNPs can be difficult to obtain for allopolyploid species with highly identical or closely related sub-genomes. For allopolyploids, due to the presence of homoeologous sequences on different sub-genomes, the SNP probes could hybridize to sequences not only on the target sub-genome, but also to the other sub-genomes or paralogs. With increasing ploidies, the fluorescent signal from a specific allele would be hard to separate from the signals of remaining alleles. Moreover, a mutation in any of the homoeologous copies may lead to the failure of probe hybridization, resulting in complex cluster types. Consequently, some attrition can be generated other than the genome-specific SNPs. Take a study applying the 90K SNP array in wheat as an example, the genotype calling revealed 35,684 (44%) assays showing three clusters, corresponding to genome-specific SNPs, 25,199 (31%) showing monomorphic clusters, and 20,704 (25%) showing complex clustering patterns due to this attrition (Wang et al., 2014).

For autopolyploids, the major complication is distinguishing between different allele dosages. However, this becomes more difficult as the ploidy increases. As SDMs only have two or three clusters, they can be easily and clearly distinguished in genotype calling for both Affymetrix and Illumina platforms. More details on SDMs are discussed in section SNP Selection for SNP Array Design and Genotype Calling in Polyploid Species above.

### General genotype calling based on Affymetrix Axiom and Illumina Infinium platforms

**Figure 1:**
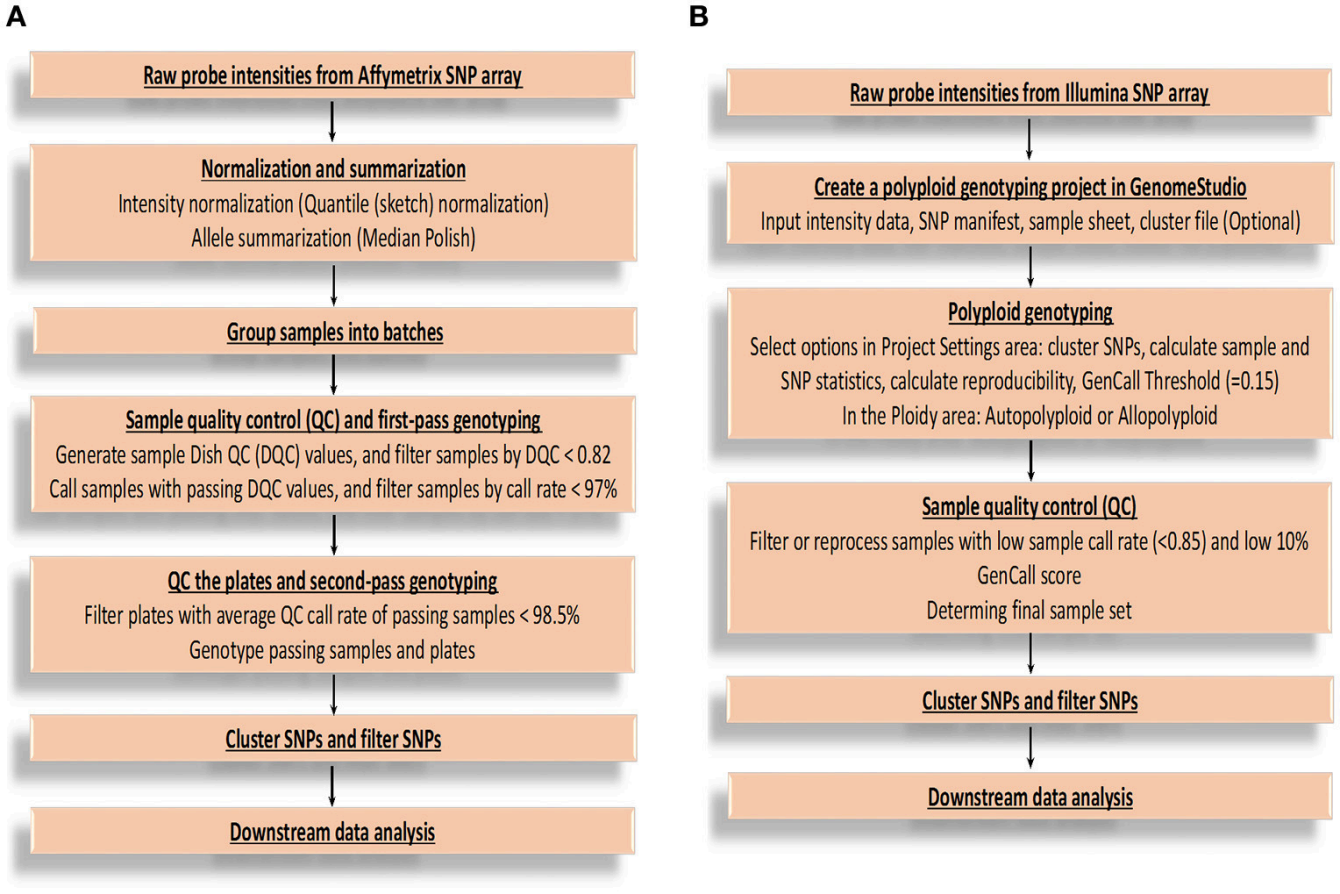
Flowcharts of genotype calling for Affymetrix and Illumina platforms. **(A)** The flowchart of executing genotype calling mainly based on Best Practices Genotyping Workflow on the Affymetrix Axiom SNP array platform. **(B)** The flowchart of executing genotype calling based on Illumina Infinium SNP array platform. GenCall score: a quality metric calculated for each genotype (data point), and ranges from 0 to 1; 10% GenCall score: 10th percentile GenCall score for all samples.

After obtaining raw probe intensities of all the SNPs for each sample in cell intensity files (CEL) from Affymetrix Axiom SNP array, there are several main steps for genotype calling (Figure 1A) by Best Practices Genotyping Workflow on this platform, starting from the normalization of raw intensities and ending at SNP clustering and filtering (Liu et al., 2017). More technical and computational details are available from Affymetrix: (http://tools.thermofisher.com/content/sfs/manuals/axiom_genotyping_solution_analysis_guide.pdf). From the report of Affymetrix, SNPs are sorted into six quality classes based on their performance of clustering, three of which report accurate genotypes and are recommended for further validation (PHR, NMH-no minor homozygote, and OTV-off target variant). The remaining three classes are not considered for further processing, because MHR (mono high resolution) cluster is monomorphic, and a simple three-cluster genotype model are not able to generate complex intensity over three clusters correctly (CRBT-call rate below threshold, and Other) (Bassil et al., 2015). Additionally, it is easy to miscall the heterozygous as homozygous especially for single-dose markers in high polyploid species when the data quality is low (Lu et al., 2013; Li et al., 2014a). This SNP calling pipeline can limit the efficiency of genotype calls for polyploid species because it is not able to call multiple allele dosages.

For Illumina Infinium genotyping data analysis, a flowchart (Figure 1B) was summarized according to GenomeStudio® Polyploid Genotyping Module v2.0 Software Guide (https://support.illumina.com/content/dam/illumina-support/documents/documentation/software_documentation/genomestudio/genomestudio-2-0/genomestudio-polyploid-genotyping-module-user-guide-1000000012407-00.pdf) and Infinium Genotyping Data Analysis (https://www.illumina.com/Documents/products/technotes/technote_infinium_genotyping_data_analysis.pdf). The steps are generally similar as that of Affymetrix except for some platform-specific parameters. The SNP genotypes are called by using GenomeStudio software based on intensity data file and SNP information file. Take allotetraploid cotton as an example (Hulse-Kemp et al., 2015), six distinct SNP classes are generated after SNP genotype calling, of which two classes of SNPs show only one genotype and thus are monomorphic SNPs. The remaining four classes of SNPs show three genotypes or clusters, which are clustered based on their GenTrain score (the SNP cluster quality score), thus are polymorphic. The SNPs from the third class, representing three genotypes (AA, AB, BB), are genome-specific markers and are recommended SNPs with reliable genotypes. The fourth class SNPs are also genome-specific markers, but assaying two homoeologous loci, one monomorphic and the other polymorphic (AAAA, AAAB, AABB). Both the fourth and fifth class SNPs require manual adjustment, which could be a challenge. The sixth class SNPs are usually thought to be failed.

### Available tools for genotype calling based on array data in polyploid species

Fortunately, three open access tools have been developed and used for genotype calling in polyploid crop species based on array data, including the R package fitTetra (Voorrips et al., 2011), superMASSA (Serang et al., 2012) (http://statgen.esalq.usp.br/SuperMASSA/), and ClusterCall (Carley et al., 2017). The R package fitTetra uses an automated method based on fitting a mixture of normal distributions. It can handle mixed ploidy with diploids and tetraploids simultaneously, where the reference diploids show the two homozygote extremes (Voorrips et al., 2011). It contains three functions, CodomMarker, fitTetra, and saveMarkerModels (Voorrips et al., 2011). The last two are written exclusively for tetraploid species, while CodomMarker function can possibly be used for other ploidies depending on the data quality (Voorrips et al., 2011; Vos et al., 2015). SuperMASSA is a web-based software with window interface (python scripts also available upon request), which implements an algorithm to find the exact maximum a posteriori (MAP) genotype configuration using a Bayesian model (Serang et al., 2012). It can also handle mixed ploidy for a diverse panel, though the ploidy of two parents should be the same for the F1 model (Garcia et al., 2013). So far, superMASSA is still the only software supporting all ploidies in genotype calling (Serang et al., 2012; Garcia et al., 2013; Balsalobre et al., 2017). Recently, Carley et al. (2017) designed an R package, ClusterCall, an automated method to convert signal intensity into different allele dosages for tetraploid genotypes which are called based on hierarchical clustering among multiple F1 populations. This process is implemented independently to each marker in two phases, training and prediction (Carley et al., 2017; Endelman et al., 2017). However, ClusterCall was designed specifically for autotetraploids.

Different genotype calling programs may perform differently on calling genotypes, because various models are implemented for each program. Given the difference of data formatting requirements from superMASSA (*x*_1_, *y*_1_) and ClusterCall (requirement of F1 population data sets), comparison of these three software programs was impractical. Therefore, to evaluate the impact of different genotype calling software on genotype calling in tetraploid as an example, the results comparing the software between fitTetra and ClusterCall were completed by Carley et al. (2017) and the results are summarized below. In addition, since superMASSA has been only successfully used in autopolyploid sugarcane, the comparison of its performance and accuracy with other tools is not available. Thus, we performed genotype calling by using the published data (intensities) from Voorrips et al. (2011) as input data for both software, superMASSA and fitTetra, and compared their results in section Comparison of Genotype Calling between fitTetra And SuperMASSA with Potato Illumina GoldenGate™ Assay Results.

### Comparison of genotype calling between fitTetra and ClusterCall with potato infinium 8303 SNP array results

#### SNP array data

Three potato F1 populations, comprised of 160, 191, and 162 progeny respectively, were genotyped with the potato Infinium 8,303 SNP array (Hamilton et al., 2011; Felcher et al., 2012). Genotype calling of these three SNP array data sets was performed by using R packages ClusterCall and fitTetra separately. For fitTetra, the saveMarkerModels function was selected, and three parameters were adjusted (p.threshold = 0.85, peak.threshold = 1, and sd.threshold = 0.1) (Carley et al., 2017). For ClusterCall, to obtain the maximum number of markers with ≥ 0.95 concordance ratio across the three potato populations, default parameter values were used (Carley et al., 2017).

#### Results of comparison

Across the three potato F1 population genotype calling results, the number of markers with over 95% concordance (the proportion of samples whose genotype was consistent with the prevalent genotype in that cluster) scored in at least one F1 population was higher when using ClusterCall (5,729 or 94.6% of the total markers) than using fitTetra (5,325, or 82.5% of its total). By increasing the threshold to one (1) concordance (perfect concordance), the number of concordant markers with ClusterCall decreased to 4,217 compared to 3,478 with fitTetra. Therefore, ClusterCall called genotypes with much higher concordance rate, thus is more accurate than fitTetra. ClusterCall used F1 populations with a large number of progeny as training data sets, which could increase the reliability of inferring genotypes based on chi-squared segregation test. In addition, ClusterCall showed higher accuracy (94.6 vs. 82.5%) and less computation time (9.5 min vs. 7.3 h) than fitTetra. However, fitTetra is dependent on fitting eight settled models and selecting the best fit by using constraints on parameters, such as the means, mixing ratios of the distributions, and Bayesian Information Criterion (BIC). Therefore, ClusterCall can improve the quality of genotype calling by using large F1 training populations, while this has no advantage for fitTetra (Voorrips et al., 2011; Vos et al., 2015; Carley et al., 2017). However, without training data of the F1 population, fitTetra would be more widely applied than ClusterCall, specifically to call genotypes from a population of large and different families or lines, as the call rate of fitTetra should improve with enough genotypes sitting in each distribution.

### Comparison of genotype calling between fitTetra and SuperMASSA with potato illumina GoldenGate™ assay results

#### SNP array data

The data set from an Illumina GoldenGate™ assay (Voorrips et al., 2011), comprised of 384 SNPs and used to genotype 224 tetraploid potato individuals, was analyzed. Three settings of fitTetra were adjusted according to Vos et al. (2015) (p.threshold = 0.95, peak.threshold = 0.99, and call.threshold = 0.60). After data filtering, out of the 86,016 data points (genotypes × individuals), 64,168 reached above criteria, and 69 of the 384 SNPs (18.0%) were rejected. Simultaneously, the same dataset from array assay was input into superMASSA software following the recommended MAP (Serang et al., 2012). The same model for genotype distribution (Hardy-Weinberg) was chosen for both softwares (Voorrips et al., 2011), with ploidyset to four.

#### Results of comparison

Using 64,168 data points from the output that survived filtering for both tools, a total of 52,364 (81.6%) common data points (a sample assigned into same cluster with same marker by both tools) were obtained. When comparing the proportion of common genotypes with respect to the total genotypes of each individual, it was noteworthy that both software programs performed better on calling the two homozygous genotypes than calling remaining heterozygous genotypes, according to the concordance rate (e.g., common homozygous/total homozygous called from a software). To be specific, the average concordance rates in calling two homozygous genotypes were 93.9% in fitTetra and 90.5% in superMASSA. However, the software became more diverged on calling three remaining heterozygous genotypes (average concordance rate 74.1% in fitTetra vs. 76.1% in superMASSA).

The Hardy-Weinberg equilibrium (HWE) model was used to assign the genotypes in both fitTetra and superMASSA, which revealed high concordance rate of genotype calls (>80% for total calls). The difference of genotype calls could be due to the usage of two different parameters to evaluate the models: smallest BIC value for fitTetra, and MAP for superMASSA. Furthermore, the common situations were described in these two software that assuming equal distance for clusters at fixed positions, which may result in assignment of samples to improper clusters (misclassification) (Grandke et al., 2016). The comparisons of the automated genotyping calling softwares are based on minimal data. To conclude on which software performs better, further validation would be needed. Checking the segregation ratio of markers in a mapping population is an important way to validate the marker genotype calling. For example, SDM are expected to segregate in 1:1 ratio in a bi-parental population. However, for high dosage markers, to have a high resolution of the segregation ratio, the population size should be big enough for this purpose. The other way could be using the NGS methods to sequence the SNP regions to a high depth and using the ratio of each haplotype allele read to determine the dosage of each SNP allele. Alternatively, the concordance genotypes can be considered as more reliable than discordance genotypes.

## Conclusion and suggestions

Although challenges exist for SNP discovery in polyploid species, progress has been made with the improved and increased number of available analysis tools (Clevenger et al., 2015; Song et al., 2016). As a high-throughput genotyping assay, SNP array technology is rightfully gaining popularity for SNP genotyping due to its flexibility, relative cost-efficiency, and automatic genotype calling, as discussed in this review.

SNP selection for SNP array design is a critical step for successful SNP array application. SNPs identified from gene enriched sequences are preferable for SNP array development (Hulse-Kemp et al., 2015; Clevenger et al., 2017). In regards to techniques, the first thing to consider for SNPs to be included in the array is whether the SNPs are suitable for probe design based on the requirements of selected platform, such as SNP depth, SNP types, SNP frequency, additional variations within probe sequence of target SNPs, and Affymetrix P-convent value or Illumina ADT value. The second consideration will be the dosage of SNPs. For polyploids such as sugarcane, given the lack of software for high dosage SNP marker analyses, SDMs could be preferred for SNP array design, as they can be treated as markers in diploids for genotype calling. Similarly, genome-specific SNPs are preferred for allopolyploids. Markers from elite cultivars, accessions and related species can be preferentially selected due to wider genetic backgrounds. To reduce the influence of ascertainment bias, the third optional consideration can be SNP distribution according to different homologous chromosomes and functions (may focus on genic regions) if the reference genome is available and fully annotated, and adding SNP resources from wild species or related species. In fact, before developing a large SNP array, a small scale of SNP array to validate some of SNPs is viable.

To call the genotypes (or dosage of each SNP locus) based on the SNP array results in polyploids, three additional open access tools are available beside the native callers from the platforms. In the only comparison of ClusterCall and fitTetra to date, ClusterCall showed higher accuracy and less computation time than fitTetra for genotype calling in an auto-tetraploid species (Carley et al., 2017). However, without training data of a F1 population, fitTetra will be more widely applied than ClusterCall, specifically for calling genotypes from a population with different families or lines. Therefore, a hybrid approach to combine ClusterCall training set calls with fitTetra prediction set calls can be applied (Carley et al., 2017). The performance of fitTetra and superMASSA were both better in calling homozygous genotypes than calling heterozygous genotypes. Currently, superMASSA is the only open-access tool that has been successfully utilized in determining ploidies and genotype calling based on SNP array data for highly polyploid species like sugarcane (Garcia et al., 2013; Costa et al., 2016; Balsalobre et al., 2017). To analyze SNP array data for highly polyploid species (ploidy >4), superMASSA software (Carley et al., 2017) with adjusted settings (default maybe not suggested for all data sets) can be used to call all the possible genotypes. An updated version of fitTetra, fitPoly, seemed to be available soon (Van Geest et al., 2017), which can be explored for calling genotypes with multiple dosages. Otherwise, the Affymetrix and Illumina genotype calling platforms are still the main choice for genotype calling of SDM or genome-specific SNPs. Hopefully, new technologies and tools will be available to analyze different allele dosages of SNP array data for highly polyploid crops such as sugarcane in the near future.

## Author contributions

JW guided the intention and outline of the review. QY prepared the manuscript draft. XY, ZP, LX, and JW critically revised and re-wrote parts of the manuscript.

### Conflict of interest statement

The authors declare that the research was conducted in the absence of any commercial or financial relationships that could be construed as a potential conflict of interest.
